# *catena*-Poly[[di-*tert*-butyl­tin(IV)]-μ-oxalato]

**DOI:** 10.1107/S160053681400539X

**Published:** 2014-03-15

**Authors:** Martin Reichelt, Hans Reuter

**Affiliations:** a Institut für Chemie neuer Materialien, Universität Osnabrück, Barbarastrasse 7, D-49069 Osnabrück, Germany

## Abstract

The title compound, [Sn(C_4_H_9_)_2_(C_2_O_4_)]_*n*_, an unexpected side product in the reaction of di-*tert*-butyl­tin(IV) oxide with nitric acid, represents the first diorganotin(IV) oxalate to be structurally characterized. The Sn^IV^ atom of the one-dimensional coordination polymer is located on a mirror plane and is coordinated by two chelating oxalate ions with two rather different Sn—O bond lengths of 2.150 (1) and 2.425 (1) Å, and two *t*-butyl groups with Sn—C bond lengths of 2.186 (2) and 2.190 (2) Å. The coordination polyhedron around the Sn^IV^ atom is a distorted tetra­gonal disphenoid. The centrosymmetric oxalate ion also has an asymmetric coordination geometry, as reflected by the two slightly different C—O bond lengths of 1.242 (2) and 1.269 (2) Å. The chains of the polymer propagate along the *b*-axis direction. Only van der Waals inter­actions are observed between the chains.

## Related literature   

For tin(II) oxalate and related compounds, see: Christie *et al.* (1979 ▶); Gleizes & Galy (1979 ▶); Ramaswamy *et al.* (2008 ▶). For (*R*_3_Sn)_2_Ox (Ox = oxalate) and related compounds, see: Diop *et al.* (2003 ▶); Ng & Kumar Das (1993 ▶); Ng *et al.* (1994 ▶); Diop *et al.* (1997 ▶). For comparative compounds, see: Reichelt & Reuter (2013 ▶).
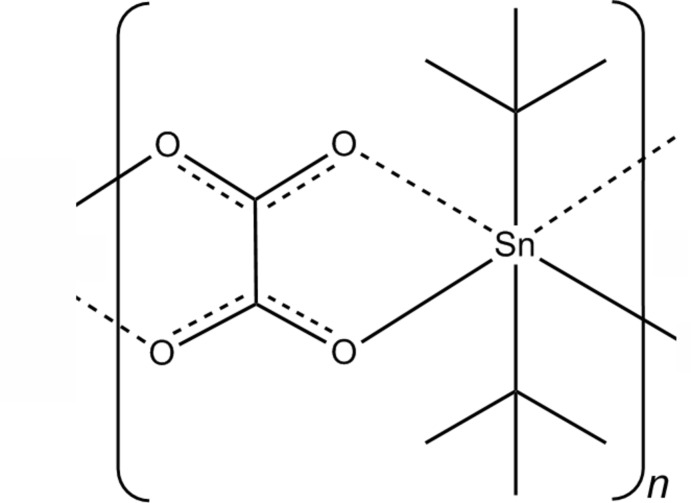


## Experimental   

### 

#### Crystal data  

[Sn(C_4_H_9_)_2_(C_2_O_4_)]
*M* *_r_* = 320.93
Orthorhombic, 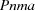

*a* = 11.5763 (3) Å
*b* = 11.3417 (3) Å
*c* = 9.3160 (2) Å
*V* = 1223.14 (5) Å^3^
*Z* = 4
Mo *K*α radiation
μ = 2.08 mm^−1^
*T* = 100 K
0.19 × 0.09 × 0.09 mm

#### Data collection  

Bruker APEXII CCD diffractometer
Absorption correction: multi-scan (*SADABS*; Bruker, 2009 ▶) *T*_min_ = 0.691, *T*_max_ = 0.843
49069 measured reflections
1548 independent reflections
1426 reflections with *I* > 2σ(*I*)
*R*_int_ = 0.081

#### Refinement  

*R*[*F* ^2^ > 2σ(*F* ^2^)] = 0.015
*wR*(*F* ^2^) = 0.038
*S* = 1.10
1548 reflections
83 parameters
H-atom parameters constrained
Δρ_max_ = 0.53 e Å^−3^
Δρ_min_ = −0.37 e Å^−3^

### 

Data collection: *APEX2* (Bruker, 2009 ▶); cell refinement: *SAINT* (Bruker, 2009 ▶); data reduction: *SAINT*; program(s) used to solve structure: *SHELXS97* (Sheldrick, 2008 ▶); program(s) used to refine structure: *SHELXL97* (Sheldrick, 2008 ▶); molecular graphics: *DIAMOND* (Brandenburg, 2006 ▶) and *Mercury* (Macrae *et al.*, 2008 ▶); software used to prepare material for publication: *SHELXTL* (Sheldrick, 2008 ▶).

## Supplementary Material

Crystal structure: contains datablock(s) I. DOI: 10.1107/S160053681400539X/cq2010sup1.cif

Structure factors: contains datablock(s) I. DOI: 10.1107/S160053681400539X/cq2010Isup2.hkl

CCDC reference: 990826

Additional supporting information:  crystallographic information; 3D view; checkCIF report

## Figures and Tables

**Fig. 1.**
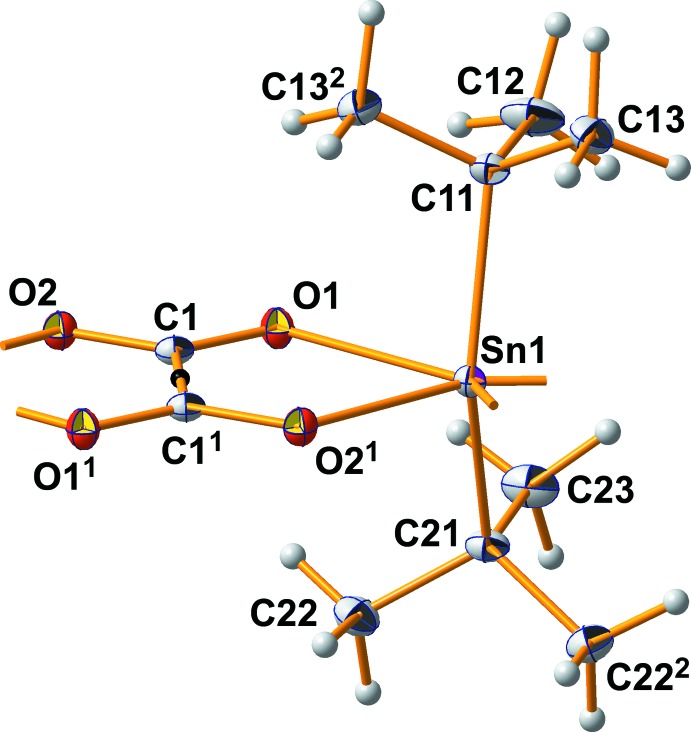
Ball-and-stick model of one formula unit in the crystal structure of di-tert-butyltin(IV) oxalate with the atomic numbering scheme used. With the exception of the hydrogen atoms, which are shown as spheres of arbitrary radius, all other atoms are drawn as thermal displacement ellipsoids of 50% probability. The hydrogen atoms of methyl groups C12 and C23 are disordered with respect to the mirror plane. The black ball indicates the centre of symmetry.

**Fig. 2.**
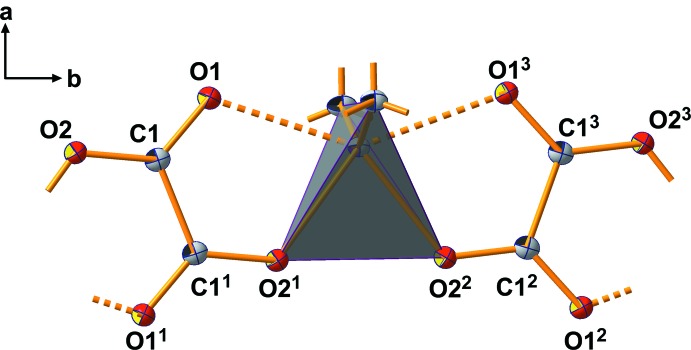
Polyhedron model of the coordination sphere of the tin atom. The tert-butyl groups have been omitted for clarity and weak Sn···O interactions are indicated by dashed sticks. Displacement ellipsoids for non-H atoms are shown with 50% probability. Symmetry transformations used to generate equivalent atoms: 1) -x, 1 - y, -z; 2) -x, 3/2 + y, -z; 3)x, 3/2 - y, z.

**Fig. 3.**
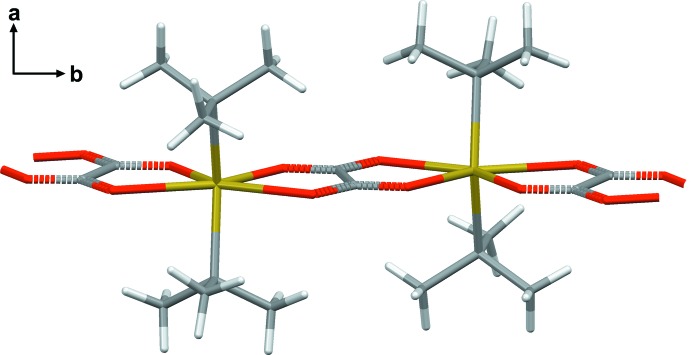
Stick model showing a part of the one-dimensional coordination polymer of the title compound, colour code: tin = bronze, oxygen = red, carbon = dark grey, hydrogen = light grey; weak interactions are drawn as dashed sticks.

**Fig. 4.**
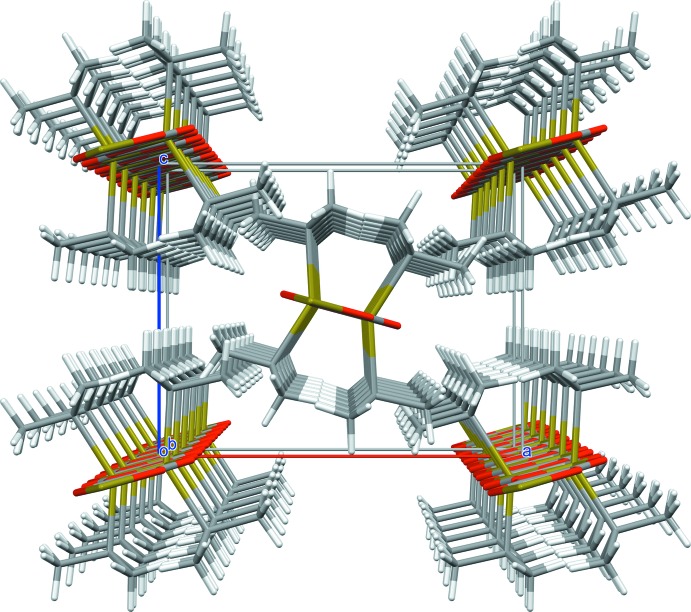
Perspective view of the crystal structure parallel to the crystallographic b axis, looking down the chains of the one-dimensional coordination polymer.

## supplementary crystallographic information

### 1.1. Synthesis and crystallization

Single crystals of di-*tert*-butyl­tin(IV) oxalate were obtained as a side product in reactions of di-*tert*-butyl­tin(IV) oxide, (*^t^*Bu_2_SnO)_3_, with nitric acid in different stoichiometric ratios. The main products were *^t^*Bu_2_Sn(NO_3_)_2_ · 2H_2_O and *^t^*Bu_2_Sn(NO_3_)(OH) · H_2_O. Larger qu­anti­ties of the title compound were obtained when 0.71 g (0.95 mmol) di-*tert*-butyl­tin(IV) oxide were stirred at ambient temperature with 6 ml nitric acid (65%, Merck), 15 ml ethanol and 20 ml water for 6 h to give a clear solution. On slow evaporation of the solvent, colourless, needle-like crystals of the title compound grew initially followed by block-like crystals of the other two compounds.

A suitable single crystal was selected under a polarization microscope and mounted on a 50 µm MicroMesh MiTeGen Micromount^TM^ using FROMBLIN Y perfluoro­polyether (LVAC 16/6, Aldrich).

### 1.2. Refinement

All hydrogen atoms could be located in difference Fourier synthesis maps. However during refinement, they were placed at idealized positions and refined whilst riding on the carbon atoms with a C—H distance of 0.98 Å and a common isotropic displacement parameter.

### 2. Comment

Oxalate ions, C_2_O_4_^2-^, Ox, play an important role as counterions or complex ligands in inorganic as well as in organometallic chemistry, not only in the chemistry of transition metals, but also in the chemistry of main group metals. This applies particularly to the *p*-block element, tin, for which many tin(II), tin(IV) and organotin(IV) oxalates are known. The main focus, however, is on anionic tin species such as [Sn^II^Ox_2_]^2-^, as found in K_2_[SnOx_2_] · H_2_O (Christie *et al.*, 1979), or [Ph_3_SnOx_2_]^-^, as found in [Me_4_N][Ph_3_SnOx_2_](Ng & Kumar Das, 1993). Structural information on pure inorganic tin(II) and tin(IV) oxalates and organotin(IV) oxalates remains rare. In case of tin(II), the structure of the oxalate, Sn(C_2_O_4_), has been described (Christie *et al.*, 1979); Gleizes & Galy, 1979) and its adducts with 2,2'-bi­pyridine and 1,10-phenanthroline (Ramaswamy *et al.*, 2008), while in case of organotin(IV) compounds, only the structures of the bis­(triorganotin(IV)) oxalates, (*R*_3_Sn)_2_Ox, (*R* = Ph, Diop *et al.*, 2003; *R* = Cy, Ng *et al.*, 1994), and of the Lewis-base stabilized bis­(aqua­tri­methyl­tin(IV)) oxalate, [Me_3_Sn(H_2_O)]_2_Ox (Diop *et al.*, 1997), have been investigated. Accordingly, the title compound represents the first diorganotin(IV) oxalate, R_2_SnOx, to be structurally characterized.

The asymmetric unit consists of half a formula unit (Fig. 1) with the centrosymmetric oxalate ion, the tin atom and both *tert*-butyl groups lying on a mirror plane. To a first approximation, the tin atom has fourfold coordination, being bonded to two *tert*-butyl groups [*d*(Sn—C) = 2.186 (2) and 2.190 (2) Å] and the two oxygen atoms of two symmetry related oxalate ions [*d*(Sn—O2) = 2.150 (1) Å]. From the bond angles of 144.29 (8)° between the *t*-butyl groups and 74.21 (6)° between the two oxygen atoms, the coordination polyhedron is compressed to a tetra­gonal disphenoid (Fig. 2).

The coordination sphere of the tin atom is completed by the other oxygen atoms, O1, of the coordinated oxalate ions that undergo a much weaker inter­action with the tin atom [*d*(Sn—O1) = 2.4245 (1) Å], resulting in a very asymmetrical bidentate coordination mode of the oxalate ions. As consequence, the C—O distances within the oxalate ion are also unequal, with the shorter one, [*d*(C1—O1) = 1.242 (2) Å], associated with the weaker coordinating oxygen atom and the longer one, [*d*(C1—O2) = 1.269 (2) Å] with the stronger coordinating oxygen atom.

The oxalate ion itself is absolutely planar as it belongs to point group *C_i_* and exhibits a C—C bond length of 1.545 (3) Å, which is slightly longer than a normal single bond between two *sp*^2^-hybridized carbon atoms. A one-dimensional coordination polymer is generated from the bilateral, side-on coordination of the oxalate ion to the organotin moieties. The chains of the polymer propagate in the direction of the crystallographic *b* axis (Fig. 3). Inter­chain inter­actions (Fig. 4) are restricted to van der Waals' ones.

Both *tert*-butyl groups have a mean value for C—C of 1.530 (3) Å [range: 1.526 (3) - 1.533 (3) Å] and a mean C—C—C angle of 109.6 (8)° [range: 110.03 (12)° - 108.09 (3)°]. Considering the Sn—C—C bond angles, both *tert*-butyl groups show similar values: two angles are around the ideal tetra­hedral value of 109.5°, whereas the third one is slightly smaller [105.7 (1)° for C21; 107.0 (2)° for C11]. Similar values were observed in the compound *^t^*Bu_2_Sn(OAc)_2_ (Reichelt & Reuter, 2013) in which the tin atoms also adopt (4 + 2) coordination geometry.

### Crystal data

**Table:**

[Sn(C_4_H_9_)_2_(C_2_O_4_)]	*F*(000) = 640
*M**_r_* = 320.93	*D*_x_ = 1.743 Mg m^−^^3^
Orthorhombic, *P**n**m**a*	Mo *K*α radiation, λ = 0.71073 Å
Hall symbol: -P 2ac 2n	Cell parameters from 9773 reflections
*a* = 11.5763 (3) Å	θ = 2.8–29.0°
*b* = 11.3417 (3) Å	µ = 2.08 mm^−^^1^
*c* = 9.3160 (2) Å	*T* = 100 K
*V* = 1223.14 (5) Å^3^	Needle, colourless
*Z* = 4	0.19 × 0.09 × 0.09 mm

### Data collection

**Table:**

Bruker APEXII CCD diffractometer	1548 independent reflections
Radiation source: fine-focus sealed tube	1426 reflections with *I* > 2σ(*I*)
Graphite monochromator	*R*_int_ = 0.081
φ and ω scans	θ_max_ = 28.0°, θ_min_ = 2.8°
Absorption correction: multi-scan (*SADABS*; Bruker, 2009)	*h* = −15→15
*T*_min_ = 0.691, *T*_max_ = 0.843	*k* = −14→14
49069 measured reflections	*l* = −12→12

### Refinement

**Table:**

Refinement on *F*^2^	Secondary atom site location: difference Fourier map
Least-squares matrix: full	Hydrogen site location: inferred from neighbouring sites
*R*[*F*^2^ > 2σ(*F*^2^)] = 0.015	H-atom parameters constrained
*wR*(*F*^2^) = 0.038	*w* = 1/[σ^2^(*F*_o_^2^) + (0.0134*P*)^2^ + 0.5948*P*] where *P* = (*F*_o_^2^ + 2*F*_c_^2^)/3
*S* = 1.10	(Δ/σ)_max_ < 0.001
1548 reflections	Δρ_max_ = 0.53 e Å^−^^3^
83 parameters	Δρ_min_ = −0.37 e Å^−^^3^
0 restraints	Extinction correction: *SHELXL97* (Sheldrick, 2008), Fc^*^=kFc[1+0.001xFc^2^λ^3^/sin(2θ)]^-1/4^
Primary atom site location: structure-invariant direct methods	Extinction coefficient: 0.0028 (3)

### Special details

**Table:**

Geometry. All e.s.d.'s (except the e.s.d. in the dihedral angle between two l.s. planes) are estimated using the full covariance matrix. The cell e.s.d.'s are taken into account individually in the estimation of e.s.d.'s in distances, angles and torsion angles; correlations between e.s.d.'s in cell parameters are only used when they are defined by crystal symmetry. An approximate (isotropic) treatment of cell e.s.d.'s is used for estimating e.s.d.'s involving l.s. planes.
Refinement. Refinement of *F*^2^ against ALL reflections. The weighted *R*-factor *wR* and goodness of fit *S* are based on *F*^2^, conventional *R*-factors *R* are based on *F*, with *F* set to zero for negative *F*^2^. The threshold expression of *F*^2^ > σ(*F*^2^) is used only for calculating *R*-factors(gt) *etc*. and is not relevant to the choice of reflections for refinement. *R*-factors based on *F*^2^ are statistically about twice as large as those based on *F*, and *R*- factors based on ALL data will be even larger.

### Fractional atomic coordinates and isotropic or equivalent isotropic displacement parameters (Å^2^)

**Table:**

	*x*	*y*	*z*	*U*_iso_*/*U*_eq_	Occ. (<1)
Sn1	0.071797 (12)	0.7500	0.025488 (15)	0.01094 (6)	
C1	0.06018 (13)	0.47568 (15)	0.02020 (16)	0.0128 (3)	
O1	0.13768 (10)	0.54788 (9)	0.04776 (11)	0.0150 (2)	
O2	0.07125 (9)	0.36437 (10)	0.02238 (12)	0.0144 (2)	
C11	0.17351 (18)	0.7500	−0.1723 (2)	0.0146 (4)	
C12	0.3005 (2)	0.7500	−0.1283 (3)	0.0286 (6)	
H12A	0.3488	0.7643	−0.2130	0.0266 (19)*	0.50
H12B	0.3204	0.6734	−0.0864	0.0266 (19)*	0.50
H12C	0.3139	0.8123	−0.0574	0.0266 (19)*	0.50
C13	0.14660 (16)	0.85947 (14)	−0.26272 (18)	0.0231 (3)	
H13A	0.1691	0.9304	−0.2096	0.0266 (19)*	
H13B	0.0636	0.8622	−0.2834	0.0266 (19)*	
H13C	0.1899	0.8558	−0.3530	0.0266 (19)*	
C21	0.08213 (18)	0.7500	0.2603 (2)	0.0150 (4)	
C22	0.02448 (15)	0.64021 (14)	0.32317 (18)	0.0207 (3)	
H22A	0.0662	0.5697	0.2914	0.0266 (19)*	
H22B	−0.0558	0.6360	0.2902	0.0266 (19)*	
H22C	0.0261	0.6445	0.4282	0.0266 (19)*	
C23	0.2108 (2)	0.7500	0.2959 (3)	0.0235 (5)	
H23A	0.2211	0.7605	0.3995	0.0266 (19)*	0.50
H23B	0.2489	0.8147	0.2448	0.0266 (19)*	0.50
H23C	0.2450	0.6748	0.2663	0.0266 (19)*	0.50

### Atomic displacement parameters (Å^2^)

**Table:**

	*U*^11^	*U*^22^	*U*^33^	*U*^12^	*U*^13^	*U*^23^
Sn1	0.01011 (9)	0.01186 (9)	0.01084 (9)	0.000	−0.00048 (5)	0.000
C1	0.0131 (8)	0.0157 (7)	0.0098 (7)	0.0009 (6)	0.0006 (5)	0.0000 (5)
O1	0.0130 (5)	0.0135 (5)	0.0184 (6)	−0.0008 (4)	−0.0020 (4)	0.0001 (4)
O2	0.0123 (6)	0.0127 (5)	0.0183 (6)	0.0006 (4)	−0.0011 (4)	0.0002 (4)
C11	0.0123 (10)	0.0180 (10)	0.0137 (10)	0.000	0.0020 (8)	0.000
C12	0.0156 (12)	0.0475 (16)	0.0228 (13)	0.000	0.0036 (10)	0.000
C13	0.0303 (9)	0.0211 (8)	0.0178 (8)	0.0007 (7)	0.0064 (7)	0.0036 (6)
C21	0.0130 (10)	0.0217 (10)	0.0103 (10)	0.000	−0.0010 (8)	0.000
C22	0.0224 (8)	0.0237 (8)	0.0159 (8)	−0.0013 (7)	0.0003 (6)	0.0031 (6)
C23	0.0142 (11)	0.0385 (13)	0.0177 (11)	0.000	−0.0034 (9)	0.000

### Geometric parameters (Å, º)

**Table:**

Sn1—O2^i^	2.150 (1)	C12—H12B	0.9800
Sn1—O2^ii^	2.150 (1)	C12—H12C	0.9800
Sn1—C11	2.186 (2)	C13—H13A	0.9800
Sn1—C21	2.190 (2)	C13—H13B	0.9800
Sn1—O1	2.425 (1)	C13—H13C	0.9800
Sn1—O1^iii^	2.425 (1)	C21—C23	1.526 (3)
C1—O1	1.2416 (19)	C21—C22^iii^	1.530 (2)
C1—O2	1.269 (2)	C21—C22	1.530 (2)
C1—C1^i^	1.545 (3)	C22—H22A	0.9800
O2—Sn1^i^	2.1503 (11)	C22—H22B	0.9800
C11—C12	1.526 (3)	C22—H22C	0.9800
C11—C13	1.533 (2)	C23—H23A	0.9800
C11—C13^iii^	1.533 (2)	C23—H23B	0.9800
C12—H12A	0.9800	C23—H23C	0.9800
			
O2^i^—Sn1—O2^ii^	74.21 (6)	H12A—C12—H12B	109.5
O2^i^—Sn1—C11	103.89 (5)	C11—C12—H12C	109.5
O2^ii^—Sn1—C11	103.89 (5)	H12A—C12—H12C	109.5
O2^i^—Sn1—C21	104.43 (5)	H12B—C12—H12C	109.5
O2^ii^—Sn1—C21	104.43 (5)	C11—C13—H13A	109.5
C11—Sn1—C21	144.29 (8)	C11—C13—H13B	109.5
O2^i^—Sn1—O1	71.92 (4)	H13A—C13—H13B	109.5
O2^ii^—Sn1—O1	146.13 (4)	C11—C13—H13C	109.5
C11—Sn1—O1	84.42 (3)	H13A—C13—H13C	109.5
C21—Sn1—O1	84.11 (3)	H13B—C13—H13C	109.5
O2^i^—Sn1—O1^iii^	146.13 (4)	C23—C21—C22^iii^	110.0 (1)
O2^ii^—Sn1—O1^iii^	71.92 (4)	C23—C21—C22	110.0 (1)
C11—Sn1—O1^iii^	84.42 (3)	C22^iii^—C21—C22	109.0 (2)
C21—Sn1—O1^iii^	84.11 (3)	C23—C21—Sn1	105.7 (1)
O1—Sn1—O1^iii^	141.95 (5)	C22^iii^—C21—Sn1	111.0 (1)
O1—C1—O2	125.4 (1)	C22—C21—Sn1	111.0 (1)
O1—C1—C1^i^	117.8 (2)	C21—C22—H22A	109.5
O2—C1—C1^i^	116.8 (2)	C21—C22—H22B	109.5
C1—O1—Sn1	112.3 (1)	H22A—C22—H22B	109.5
C1—O2—Sn1^i^	121.3 (1)	C21—C22—H22C	109.5
C12—C11—C13	110.1 (1)	H22A—C22—H22C	109.5
C12—C11—C13^iii^	110.1 (1)	H22B—C22—H22C	109.5
C13—C11—C13^iii^	108.2 (2)	C21—C23—H23A	109.5
C12—C11—Sn1	107.0 (2)	C21—C23—H23B	109.5
C13—C11—Sn1	110.7 (1)	H23A—C23—H23B	109.5
C13^iii^—C11—Sn1	110.7 (1)	C21—C23—H23C	109.5
C11—C12—H12A	109.5	H23A—C23—H23C	109.5
C11—C12—H12B	109.5	H23B—C23—H23C	109.5

Symmetry codes: (i) −*x*, −*y*+1, −*z*; (ii) −*x*, *y*+1/2, −*z*; (iii) *x*, −*y*+3/2, *z*.

## References

[bb1] Brandenburg, K. (2006). *DIAMOND* Crystal Impact GbR, Bonn, Germany.

[bb2] Bruker (2009). *APEX2*, *SADABS* and *SAINT* Bruker AXS Inc., Madison, Wisconsin, USA.

[bb3] Christie, A. D., Howie, R. A. & Moser, W. (1979). *Inorg. Chim. Acta*, **36**, L447–L448.

[bb4] Diop, L., Mahieu, B., Mahon, M. F., Molloy, K. C. & Okio, K. Y. A. (2003). *Appl. Organomet. Chem.* **17**, 881–882.

[bb5] Diop, L., Mahon, M. F., Molloy, K. C. & Sidibe, M. (1997). *Main Group Met. Chem.* **20**, 649–654.

[bb6] Gleizes, A. & Galy, J. (1979). *J. Solid State Chem.* **30**, 23–33.

[bb7] Macrae, C. F., Bruno, I. J., Chisholm, J. A., Edgington, P. R., McCabe, P., Pidcock, E., Rodriguez-Monge, L., Taylor, R., van de Streek, J. & Wood, P. A. (2008). *J. Appl. Cryst.* **41**, 466–470.

[bb8] Ng, S. W. & Kumar Das, V. G. (1993). *J. Organomet. Chem.* **456**, 175–179.

[bb9] Ng, S. W., Kumar Das, V. G., Li, S.-L. & Mak, T. C. W. (1994). *J. Organomet. Chem.* **467**, 47–49.

[bb10] Ramaswamy, P., Datta, A. & Natarajan, S. (2008). *Eur. J. Inorg. Chem.* pp. 1376–1385.

[bb11] Reichelt, M. & Reuter, H. (2013). *Acta Cryst.* E**69**, m254. 10.1107/S1600536813009185 PMC3647801 23723767

[bb12] Sheldrick, G. M. (2008). *Acta Cryst.* A**64**, 112–122. 10.1107/S0108767307043930 18156677

